# Characteristics of Hf_0.5_Zr_0.5_O_2_ Thin Films Prepared by Direct and Remote Plasma Atomic Layer Deposition for Application to Ferroelectric Memory

**DOI:** 10.3390/nano13050900

**Published:** 2023-02-27

**Authors:** Da Hee Hong, Jae Hoon Yoo, Won Ji Park, So Won Kim, Jong Hwan Kim, Sae Hoon Uhm, Hee Chul Lee

**Affiliations:** 1 Department of Advanced Materials Engineering, Tech University of Korea, Siheung 15073, Republic of Korea; 2 EN2CORE Technology Inc., Daejeon 18469, Republic of Korea

**Keywords:** HZO, PEALD, remote plasma, ferroelectric, antiferroelectric, fatigue endurance

## Abstract

Hf_0.5_Zr_0.5_O_2_ (HZO) thin film exhibits ferroelectric properties and is presumed to be suitable for use in next-generation memory devices because of its compatibility with the complementary metal–oxide–semiconductor (CMOS) process. This study examined the physical and electrical properties of HZO thin films deposited by two plasma-enhanced atomic layer deposition (PEALD) methods— direct plasma atomic layer deposition (DPALD) and remote plasma atomic layer deposition (RPALD)—and the effects of plasma application on the properties of HZO thin films. The initial conditions for HZO thin film deposition, depending on the RPALD deposition temperature, were established based on previous research on HZO thin films deposited by the DPALD method. The results show that as the measurement temperature increases, the electric properties of DPALD HZO quickly deteriorate; however, the RPALD HZO thin film exhibited excellent fatigue endurance at a measurement temperature of 60 °C or less. HZO thin films deposited by the DPALD and RPALD methods exhibited relatively good remanent polarization and fatigue endurance, respectively. These results confirm the applicability of the HZO thin films deposited by the RPALD method as ferroelectric memory devices.

## 1. Introduction

The effects of various doping elements, such as Y, Al, La, and Zr, on the ferroelectric properties of HfO_x_-based thin films have been reported [1,2,3,4,5,6]. HfO_x_-based Hf_x_Zr_1−x_O_2_ thin films doped with Zr by the atomic layer deposition (ALD) method exhibit paraelectric, antiferroelectric, and ferroelectric properties, depending on the composition and crystal structure of the monoclinic (m), tetragonal (t), or orthorhombic (o) phase [7,8,9,10,11]. Hf_0.5_Zr_0.5_O_2_ (HZO) thin film deposited at a 1:1 ratio has ferroelectric properties, even at a low thickness of several nm, and is presumed to be suitable for use in next-generation memory devices because of its compatibility with the complementary metal–oxide–semiconductor (CMOS) process [7,8,9,10,11,12,13,14,15,16]. However, the use of HZO thin film as a next-generation memory device requires the improvement of its electrical properties, including its remanent polarization (P_r_) and fatigue endurance, through the improvement of its thin film properties, such as its crystal structure and reduction in oxygen defects [17,18,19,20]. Extensive research has been conducted on ferroelectric HZO thin films to achieve a high ratio of o-phase, which is known to exhibit ferroelectricity, although it has low durability. Recent studies have shown that antiferroelectric HZO thin films show lower P_r_ values, but better durability than ferroelectric HZO [12,21,22,23,24].

The most widely used plasma-enhanced atomic layer deposition (PEALD) method for HZO thin film deposition is direct plasma ALD (DPALD), in which plasma is applied directly onto the substrate. It forms ions and radicals of the reaction gas, increasing the reactivity with the precursor and widening the process window. As a result, DPALD offers the advantage of a high deposition rate by enabling the deposition of high-density and high-quality thin films, even at relatively low deposition temperatures [25,26,27]. However, prior studies have reported that even low-power plasma applied for a short period of time affects the interface and electric properties of the semiconductor because of the activation of surface reaction and negative charge [28,29,30,31]. The remote plasma ALD (RPALD) method separates the plasma generation space and the deposition process space and utilizes radicals generated from the plasma. Because the RPALD method uses highly reactive radicals, deposition can be performed at an even lower temperature than that of thermal ALD, which uses heat as an activation energy source. In addition, it is expected to minimize damage by direct application of plasma onto the substrate, as does DPALD [32]. However, the process conditions must be optimized to obtain a thin film with desired properties because the survival time of the radicals generated by plasma is extremely short [27,33]. Furthermore, although single thin film deposition using the RPALD method has been extensively researched, studies on the deposition and durability of HZO composite thin films are scarce [28,34,35].

Therefore, the initial conditions for HZO thin film deposition, depending on the RPALD deposition temperature, were established in this study based on previous research on HZO thin films deposited by the DPALD method [36]. Next, the process conditions were optimized by comparing the crystallinity and microstructure of the HZO thin films deposited at the same temperature using the DPALD and RPALD methods in terms of the heat treatment temperature and duration. Finally, the P–E hysteresis loop and fatigue endurance properties of the HZO thin film, heat-treated under optimized conditions, were evaluated in terms of the measurement temperature. The effects of the plasma application method and the measurement temperature on the electrical properties of the HZO thin film were then examined.

## 2. Materials and Methods

### 2.1. HZO Thin Film Deposition by DPALD and RPALD Methods

**Figure 1:**
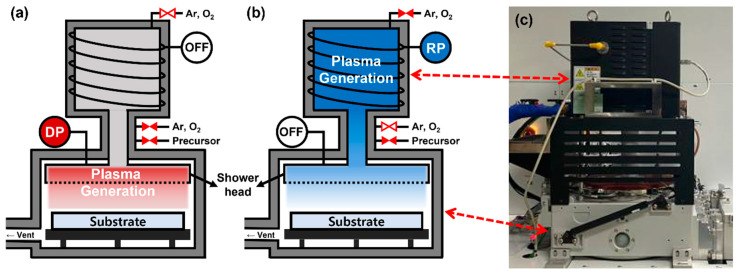
Concept diagrams of (**a**) DPALD and (**b**) RPALD processes, according to the plasma application method used in this study, and (**c**) photograph of the actual equipment.

The PEALD equipment for HZO thin film deposition and the plasma application method, according to the deposition process, are illustrated in Figure 1. As shown in Figure 1a, the DPALD method uses PEALD equipment (iOV-dx2, iSAC Research, Daejeon, Korea). For the RPALD method, a remote plasma system (En2ra-RPS, EN2CORE technology, Daejeon, Korea) was installed, in addition to the existing PEALD equipment used for deposition, as shown in Figure 1b. Reactively sputtered 50 nm thick TiN film on a SiO_2_ (100 nm)/Si (p-type) is used as the bottom electrode for HZO thin film deposition. The resistivity of the Si wafer is in the range of 1–30 Ω⋅cm. The SiO_2_ buffer layer prevents the reaction between the Si substrate and the TiN bottom electrode. Tetrakis(ethylmethylamino)-hafnium (TEMAH, iChems, Hwaseong, Korea) and Tetrakis(ethylmethylamino)-zirconium (TEMAZ, iChems, Hwaseong, Korea) were used as precursors of HfO_2_ and ZrO_2_.

First, experiments were conducted to optimize the characteristics of HfO_2_ and ZrO_2_ single thin films, while changing the deposition temperature and source dose time. Then, an HZO composite thin film was deposited at a thickness of 10 nm by alternating HfO_2_ and ZrO_2_ at a 1:1 ratio under the optimized single thin film process conditions, as shown in Table 1. One big difference between DPALD and RPALD is the method of supplying the reactant, as shown in Table 1. The DPALD method formed oxide through the plasma power of 200 W after O_2_ reactant gas was supplied, as shown in Table 1a. In the RPALD method, high-density plasma was ignited with a power of 2.6 kW in an Ar atmosphere; subsequently, O_2_ reactant gas was injected through the remote plasma, as shown in Table 1b. Optimal oxidant flow and reaction time varies from study to study [37,38]. In this study, suitable conditions for the sufficient oxidation reaction for DPALD and RPALD were obtained through previous experiments, as shown in Table 1. A metal–ferroelectrics–metal (MFM) device with TiN–HZO–TiN structure was fabricated by forming a circular TiN top electrode, with a diameter of 100 μm and a thickness of 50 nm, using RF magnetron sputtering and lift-off patterning. Subsequently, the HZO thin film was crystallized by rapid thermal annealing (RTA) in a temperature range of 500 to 800 °C and a time range of 30 to 60 s.

### 2.2. Evaluation of the Properties of DP HZO and RP HZO Thin Films

The thickness and refractive index of the deposited single thin films were measured using an ellipsometer (Elli-SE, Ellipso Technology, Suwon, Korea), and the crystallinity before and after heat treatment was examined using high-resolution X-ray diffraction (HR-XRD; Smartlab, Rigaku, Tokyo, Japan). The cross-section image, thickness, and crystallinity of the HZO thin film deposited by the DPALD and RPALD methods were measured using field-emission transmission electron microscopy (FE-TEM; Tecnai G2 F20, FEI, Hillsboro, OR, USA). The composition and chemical bonding conditions of the thin films were analyzed using X-ray photoelectron spectroscopy (XPS; AXIS-NOVA, Manchester, UK). The electrical properties of the P–E curve and the fatigue endurance of the thin films were evaluated in 20 °C intervals in the range of 20–100 °C using a semiconductor characterization system (4200A-SCS, Keithley, Cleveland, OH, USA) connected to a Peltier chuck system (DTS-P200, WIT, Suwon, Korea) and a microprobe station (APX-6B, WIT, Suwon, Korea). The P–E hysteresis loop curves were measured at the frequency of 1 kHz, with a triangle pulse of ±3V. Accordingly, the sweeping rate was approximately ±12,000 V/s. HZO thin films are known to exhibit a phenomenon called the wake-up effect. The wake-up effect refers to the stable polarization characteristics after some device cycling [39]. In this work, the P–E hysteresis loop curve was obtained after 10^5^ cycles. The fatigue endurance was measured by applying a 10-kHz square pulse of ±3 V, and the P–E hysteresis loop was measured in the specified cycles up to 10^8^ cycles.

## 3. Results and Discussion

**Figure 2:**
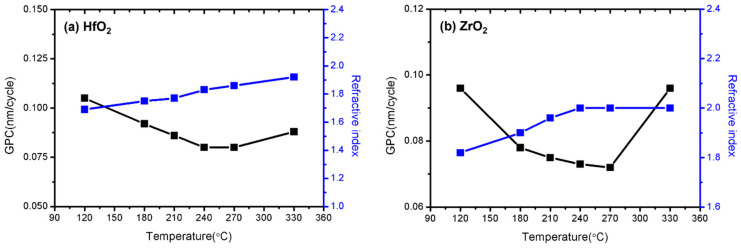
Changes in the GPC and refractive index of the (**a**) HfO_2_ and (**b**) ZrO_2_ single thin films deposited by RPALD, according to the deposition temperature.

Figure 2 shows changes in the growth per cycle (GPC) and refractive index of each single thin film of HfO_2_ and ZrO_2_ deposited by the RPALD method in the temperature range of 120 to 330 °C. The GPC error was within 0.006 nm/cycle in the range of 210–270 °C for the HfO_2_ thin film and 180–270 °C for the ZrO_2_ thin film, and this section was defined as the ALD window. Based on the above results, 210–270 °C was predicted as the common ALD window for depositing HZO composite thin films. The HfO_2_ single film approached the bulk refractive index value of 1.98 as the temperature increased from 210 °C. However, the ZrO_2_ thin film showed the smallest error, with the bulk refractive index value of 2.02 at 240 °C, and the refractive index decreased as the deposition temperature increased [40]. As noted in our previous study, the refractive index of the HfO_2_ and ZrO_2_ films is related to the film density, according to the Lorentz–Lorenz relation [36]. This relationship means that the density of the dielectric films gradually increases as the refractive index increases. The following experiments were performed at 240 °C, which is the middle of the ALD window temperature range.

**Figure 3:**
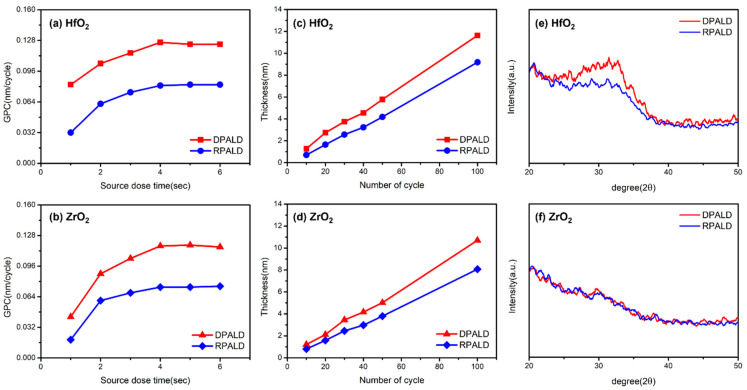
HfO_2_ and ZrO_2_ single thin films deposited by two PEALD methods: (**a**,**b**) GPC for the source time, (**c**,**d**) thickness according to the number of cycles, and (**e**,**f**) XRD patterns immediately after deposition.

Figure 3a,b shows the GPC values that change according to the source dose time of the HfO_2_ and ZrO_2_ single thin films deposited by the two PEALD methods. The GPCs of all four single thin films increased until the source dose time of 4 s, when they were then saturated. These results indicate that the source dose time of the process was set to 5 s. Figure 3c,d shows the changes in the thicknesses of single thin films according to the number of cycles. The DPALD and RPALD methods confirmed that the thickness of the HfO_2_ and ZrO_2_ thin films increased linearly with the number of deposition cycles. Furthermore, all four straight lines passed through the origin when extended; thus, the initial substrate effect was hardly shown. Figure 3a–d shows the self-limiting characteristics of ALD, and the deposition rate of the DPALD method was higher than that of the RPALD method. Figure 3e,f shows the XRD patterns of HfO_2_ and ZrO_2_ single films deposited by the DPALD and RPALD methods before heat treatment. In the case of a single HfO_2_ thin film, it was confirmed that there was a difference in crystallinity, depending on the deposition method. Since the peak intensity of the HfO_2_ thin film deposited by the DPALD method is higher than that of the RPALD method, it is considered that the crystallization is better. In contrast, the ZrO_2_ thin film showed the same non-crystallized pattern, even though the deposition method was different. Based on the results in Figure 3, the HfO_2_ and ZrO_2_ thin films deposited by the DPALD method at a deposition temperature of 240 °C had GPC values of 0.124 nm/cycle and 0.118 nm/cycle, respectively. In addition, the HfO_2_ and ZrO_2_ thin films deposited by the RPALD method showed GPC values of 0.082 nm/cycle and 0.074 nm/cycle, respectively. Based on each GPC, two thin films were deposited alternately for 42 cycles with the DPALD method, and 64 cycles with the RPALD method, to deposit about 10 nm HZO thin films at a 1:1 ratio.

**Figure 4:**
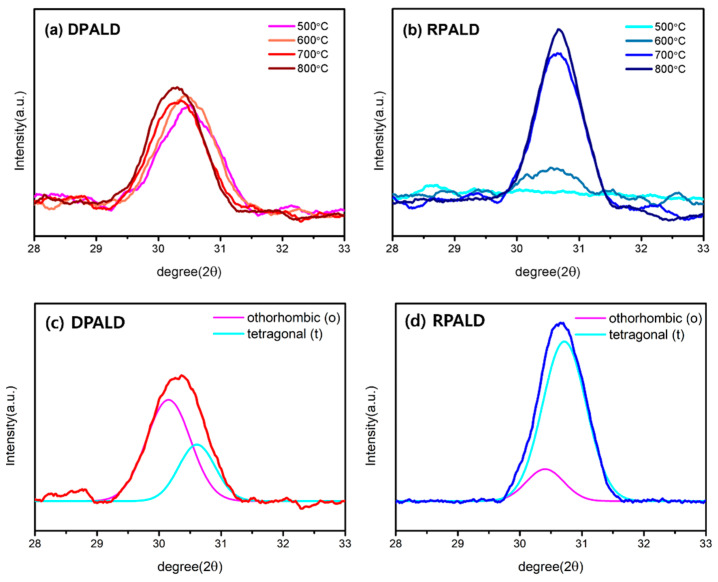
HZO thin films deposited by two PEALD methods: (**a**,**b**) XRD patterns, according to the RTA temperature, and (**c**,**d**) peak separation of the XRD pattern for heat treatment at the RTA temperature of 700 °C.

Figure 4a,b shows changes in the XRD patterns of the HZO thin films deposited by the two methods, according to the RTA temperature of 500–800 °C. Figure 4a confirmed that the HZO thin film deposited by the DPALD method was crystallized in the entire RTA temperature range. Furthermore, the peak moved in the direction of the low diffraction angle as the heat treatment temperature increased. Figure 4b shows that the HZO thin film deposited by the RPALD method was sufficiently crystallized only at relatively high temperatures of 700 °C and 800 °C. Only the peak intensity increased, and no peak shift was observed. The occurrence of sufficient crystallization of the HZO thin film deposited by the RPALD method only at a high RTA temperature can be attributed to the initial crystallinity of the HfO_2_ single thin film, as shown in Figure 3e. Evidently, the HfO_2_ thin film deposited by the RPALD method was insufficiently crystallized as compared to the HfO_2_ thin film deposited by the DPALD method. This indicates that RPALD HZO requires more energy for sufficient crystallization. Figure 4c,d show the ratio of the o- and t-phases by XRD peak separation of HZO thin films deposited by each method and then heat-treated at 700 °C. Assuming that there is little stress and consequent strain, each XRD peak was separated using the Gaussian deconvolution method [41]. The HZO thin film deposited by the DPALD method showed that the o-phase was dominant over the t-phase. In contrast, the intensity of the t-phase was much stronger than that of the o-phase in the HZO thin film deposited by the RPALD method.

**Figure 5:**
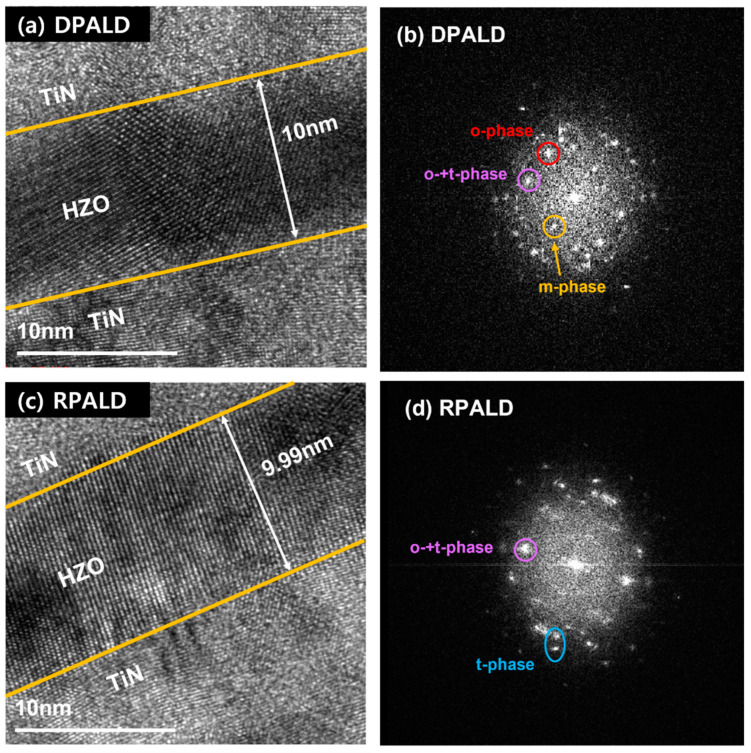
Cross-sectional TEM images and FFT patterns of HZO thin films deposited by (**a**,**b**) DPALD and (**c**,**d**) RPALD methods after heat treatment at 700 °C.

The TEM images and fast Fourier transform (FFT) patterns of HZO thin films deposited by the DPALD and RPALD methods are shown in Figure 5. The fabricated MFM device structure is top TiN/HZO/bottom TiN/SiO_2_/Si. Figure 5a and c shows the HZO thin film deposited between the top and bottom TiN electrodes using the super-cycle method, with 42 cycles of DPALD and 64 cycles of RPALD, respectively. They had a thickness of 10 nm under the deposition conditions of HfO_2_ and ZrO_2_ single thin films in regards to each PEALD method. Figure 5b and d shows the patterns of FFT performed, together with cross-sectional TEM image analysis, showing that sufficient crystallization occurred after heat treatment at 700 °C for both thin films deposited by the two methods. Moreover, the observed patterns indicate a mixture of the o-phase and t-phase in the case of both thin films. The HZO thin film deposited by the DPALD method showed the o-phase, with a lattice constant of 2.94 Å, as well as the m-phase and t-phase [42,43]. In contrast, in the HZO thin films deposited by the RPALD method, the m-phase was rarely observed, and the t-phase with a lattice constant of 2.52 Å was observed along with the o-phase [44,45].

**Figure 6:**
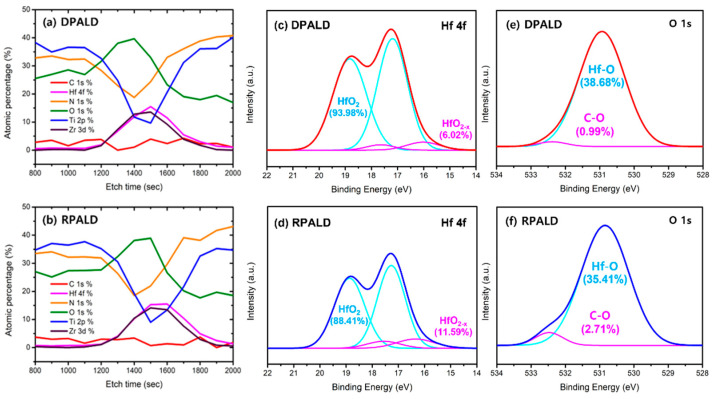
HZO thin films deposited by DPALD and RPALD methods: (**a**,**b**) XPS depth profiling, (**c**,**d**) Hf 4f, and (**e**,**f**) O 1s narrow scan XPS patterns.

XPS analysis was performed to find the cause of the differences in crystallinity and crystal structure resulting from the deposition methods, even at the same deposition and heat treatment temperatures. The XPS depth profiling in Figure 6a,b indicates that there was almost no difference in the C 1s atomic percentage of the HZO thin films deposited by the two methods. The C element in the HZO films could be attributed to an insufficient reaction of the carbon-containing precursors and the oxidant. The atomic ratios of Hf and Zr deposited were 0.53:0.47 for the DPALD method and 0.52:0.48 for the RPALD method, which are similar. In addition, Hf 4f and O 1s narrow scans were performed. Figure 6c,d shows Hf 4f narrow scan XPS patterns of the HZO thin film deposited by each method. These two patterns suggest that the HZO thin film deposited by the RPALD method has a lower stoichiometric HfO_2_ peak and a higher ratio of non-stoichiometric HfO_2-x_ peak than those deposited by the DPALD method [46,47,48]. Figure 6e,f shows the results of the O 1s narrow scan XPS patterns of the HZO thin film. Hf–O denotes the ratio of oxygen combined with Hf, and C–O denotes oxygen combined with carbon. According to previous literature, the relative value of oxygen vacancy is correlated to the C–O peak of the O 1s narrow scan [49,50]. As a result, the C–O bonding ratio is proportional to the oxygen vacancy content. It was found that the C–O bonding ratio of the HZO thin film deposited by the RPALD method was higher than that of the HZO thin film deposited by the DPALD method. The above results suggest that more oxygen vacancy exists in the RPALD HZO thin film with a higher C–O bonding ratio.

**Figure 7:**
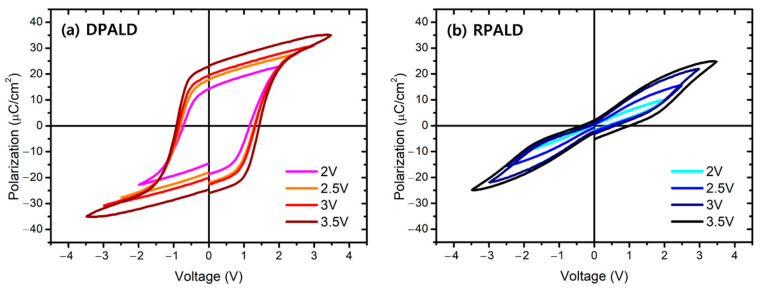
P–E hysteresis loop curves of the HZO thin films deposited by the (**a**) DPALD and (**b**) RPALD methods, according to the measurement voltage after 10^5^ cycles.

Figure 7 shows the P–E curves of HZO thin films deposited by the two methods, measured in the voltage range of ±2 to 3.5 V. The XRD patterns shown in Figure 4 indicate that the HZO thin film deposited by the o-phase-dominant DPALD method exhibited ferroelectricity, whereas the HZO thin film deposited by the t-phase-dominant RPALD method exhibited antiferroelectricity. When the measured voltage was ±3 V, the DPALD HZO had a 2P_r_ value of up to 42.62 μC/cm^2^ at 0 V, and the RPALD HZO had a 2P_r_ value of 2.99 μC/cm^2^ at 0 V. When the voltage was shifted to 1.5 V, the maximum 2P_r_ value was 7 μC/cm^2^ [23]. The significant x-axis shift of P–E hysteresis in the DPALD HZO film may be due to the formation of asymmetric interfaces. The top and bottom interfaces of the HZO thin films experience different ambient environments. In particular, the bottom TiN electrode is directly exposed to the plasma containing oxygen radicals and can easily oxidize. [51]. The polarization of the RPALD HZO film was much smaller than that of the DPALD HZO samples. However, 7 μC/cm^2^ is a sufficient value to distinguish between logical “1” and logical “0” as a ferroelectric memory [52].

**Figure 8:**
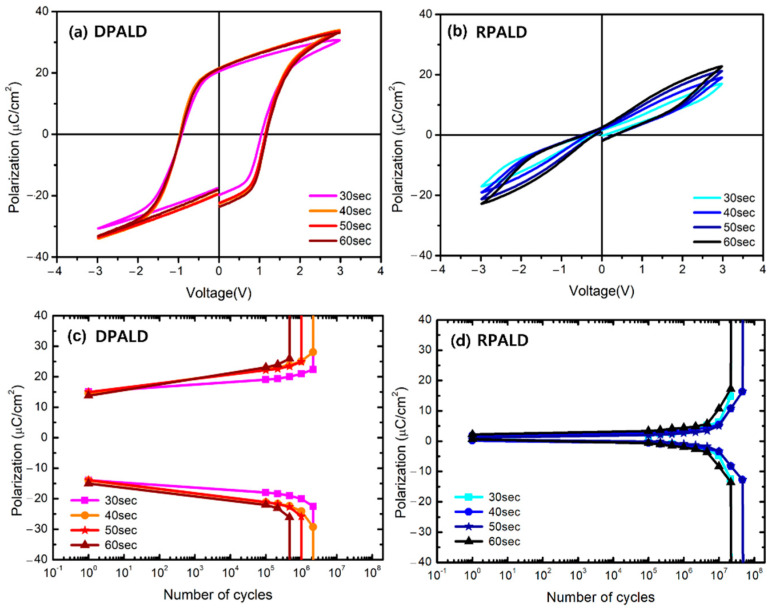
(**a**,**b**) The P–E hysteresis loop curve after 10^5^ cycles and (**c**,**d**) the fatigue endurance property of HZO thin films deposited by two PEALD methods, according to the RTA time.

Figure 8 shows the measurement results of electrical properties according to the RTA time of the HZO thin films deposited by the two PEALD methods. Figure 8a,b show the changing P–E curves of the HZO thin films deposited at 700 °C using the DPALD and RPALD methods, according to the RTA time. The remanent polarization value of the HZO thin film deposited by the DPALD method increased as the RTA time increased from 30 s to 40 s. In the range of 40 to 60 s, the remanent polarization value did not increase significantly, and the P–E curve showed a constant form. In contrast, the HZO thin film deposited by the RPALD method showed a gradual increase in 2P_r_ value as the RTA time increased. Figure 8c,d shows the fatigue endurance properties according to the RTA time of the HZO thin films deposited by the two PEALD methods. The HZO thin film deposited by the DPALD method had a lifespan ranging from 10^5^ to 10^6^ cycles. Similar lifespans were shown for the RTA time of up to 40 s; however, the lifespan decreased as the heat treatment time increased. However, the HZO thin film deposited by the RPALD method showed an excellent fatigue endurance property of more than 10^7^ cycles and exhibited a lifespan longer than 10^7^ cycles, even when the RTA time increased to 60 s. Despite the same composition, deposition temperature, and heat treatment conditions, the crystallinity, P–E curve, and fatigue endurance varied according to the deposition method. This is attributable to the amount of oxygen vacancy in the thin film [53,54]. According to previous studies, an appropriate amount of oxygen vacancy helps the formation of ferroelectric o-phase. However, the appropriate amount of oxygen vacancy required to obtain the o-phase has not been found. Thus, it is presumed that an antiferroelectric t-phase was formed because the formation and growth of the ferroelectric domain were inhibited when the oxygen vacancy exceeded the appropriate amount [55,56,57].

**Figure 9:**
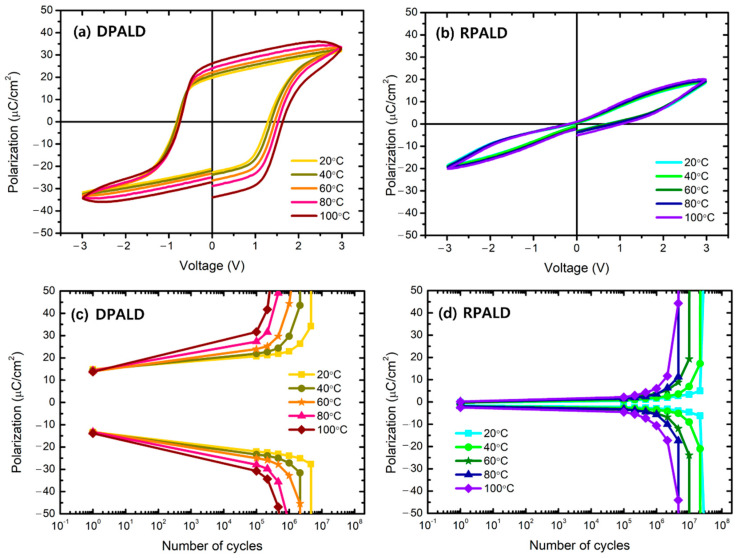
(**a**,**b**) The P–E hysteresis loop curve after 10^5^ cycles and (**c**,**d**) the fatigue endurance property of HZO films deposited by DPALD and RPALD methods, according to the measurement temperature.

Figure 9 shows the results of the electrical properties of the HZO thin films deposited by two PEALD methods in the range of 20 to 100 °C, measured to investigate why RPALDs with more oxygen vacancy exhibit better fatigue endurance. Figure 9a shows that as the measurement temperature increased from 20 °C to 100 °C, the c, d leakage current of the HZO thin film deposited by the DPALD method increased, thus changing the shape of the P–E curve. In contrast, the HZO thin film deposited by the RPALD method hardly changed with the measurement temperature, as shown in Figure 9b. The difference between these two thin films can be observed more clearly in the fatigue endurance property. Figure 9c,d shows the fatigue endurance property, according to the measured temperature of the HZO thin films deposited by the two methods. The lifespan of the DPALD HZO thin film decreased as the measured temperature increased, indicating that the thin film was considerably affected by the measured temperature. However, the RPALD HZO thin film maintained a fatigue endurance of more than 10^7^ cycles in the temperature range of 20–60 °C. Moreover, the lifespan of the thin film decreased at high temperatures of 80 to 100 °C; however, the lifespan was longer than that of DPALD HZO. It was presumed that the low dependence of the fatigue endurance on the temperature of the HZO thin film deposited by the RPALD method was caused by the low mobility of oxygen vacancy in the thin film. This means that more energy is required to move oxygen vacancies in the RPALD films. It has been found that regarding the fatigue endurance property, breakdown at the end of life occurs when the oxygen vacancy moves to the interface of the HZO thin film, piles up, and forms a large space charge [58]. The HZO thin films deposited by the RPALD method possess a greater oxygen vacancy than those deposited by the DPALD method, but show better fatigue endurance because their movement is limited by the domain interface, even though they receive thermal energy [59].

## 4. Conclusions

This study evaluated the physical and electrical properties of HZO thin films deposited by two PEALD methods—DPALD and RPALD—and examined the effects of plasma application on the properties of HZO thin films. HZO thin films with a thickness of 10 nm and an HfO_2_-to-ZrO_2_ ratio of 1:1 were deposited using the super-cycle method under deposition conditions such that each single thin film had a constant GPC. HZO thin films deposited by the two methods had a combination of o-phase and t-phase. As a result of electrical characterization, the DPALD HZO thin film was found to exhibit ferroelectric characteristics, with a 2P_r_ value of up to 42.62 μC/cm^2^, dominated by the o-phase, and a lifespan of 10^5^ to 10^6^ cycles. In contrast, the RPALD HZO thin film exhibited antiferroelectric characteristics, with a 2P_r_ value of up to approximately 7 μC/cm^2^, dominated by the t-phase, and excellent fatigue endurance of more than 10^7^ cycles. To identify the cause of the excellent fatigue endurance characteristics, the electrical properties were evaluated with respect to the measurement temperature. The results show that as the measurement temperature increases, the electrical properties of DPALD HZO quickly deteriorate; however, the RPALD HZO thin film exhibits fatigue endurance of more than 10^7^ cycles at a measurement temperature of 60 °C or less. Consequently, the HZO thin films deposited by the DPALD and RPALD methods exhibited relatively good remanent polarization and fatigue endurance characteristics, respectively. These results confirm the applicability of the HZO thin films deposited by the RPALD method as ferroelectric memory devices.

## Figures and Tables

**Table 1:** Process conditions of (**a**) DPALD and (**b**) RPALD methods and a recipe for one cycle (deposition reference temperature 240 °C).

(a) DPALD			(b) RPALD		
TEMA-(Hf, Zr) 1:1 cycle		Pressure: 1.3 Torr	TEMA-(Hf, Zr) 1:1 cycle		Pressure: 1.7 Torr
**Source**	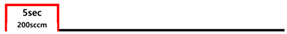		**Source**	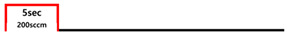	
**Ar** **Purge**	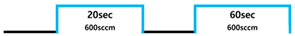		**Ar** **Purge**	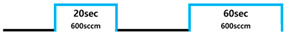	
**RF** **Plasma**	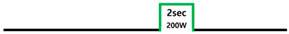		**RF** **Plasma**	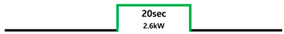	
**O_2_** **Reactant**	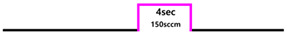		**O_2_** **Reactant**	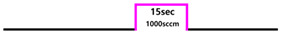	

## Data Availability

The data presented in this study are contained within the article.
